# AMG-510 and cisplatin combination increases antitumor effect in lung adenocarcinoma with mutation of KRAS G12C: a preclinical and translational research

**DOI:** 10.1007/s12672-023-00698-z

**Published:** 2023-06-07

**Authors:** Lei-Lei Wu, Wen-Mei Jiang, Zhi-Yuan Liu, Yi-Yi Zhang, Jia-Yi Qian, Yu’e Liu, Yang-Yu Huang, Kun Li, Zhi-Xin Li, Guo-Wei Ma, Dong Xie

**Affiliations:** 1 Department of Thoracic Surgery, Shanghai Pulmonary Hospital, School of Medicine, Tongji University, Shanghai, 200433 People’s Republic of China; 2 State Key Laboratory of Oncology in South China, Collaborative Innovation Center for Cancer Medicine, Sun Yat-sen University Cancer Center, Guangzhou, 510030 People’s Republic of China; 3 School of Medicine, Tongji University, Shanghai, 200092 People’s Republic of China; 4 Faculty of Biology, Medicine and Health, School of Biological Sciences, The University of Manchester, Manchester, UK

**Keywords:** KRAS G12C mutation, AMG-510, Cisplatin, In vivo, Translational medicine

## Abstract

**Background:**

The efficacy of monotherapy of AMG-510 is limited. This study explored whether the AMG-510 and cisplatin combination increases the anti-tumor effect in lung adenocarcinoma with the mutation of Kirsten rat sarcoma viral oncogene (KRAS) G12C.

**Methods:**

Patients’ data were used to analyze the proportion of KRAS G12C mutation. Besides, the next-generation sequencing data was used to uncover information about co-mutations. The cell viability assay, the concentration inhibiting 50% of cell viability (IC50) determination, colony formation, and cell-derived xenografts were conducted to explore the anti-tumor effect of AMG-510, Cisplatin, and their combination in vivo. The bioinformatic analysis was conducted to reveal the potential mechanism of drug combination with improved anticancer effect.

**Results:**

The proportion of KRAS mutation was 2.2% (11/495). In this cohort with KRAS mutation, the proportion of G12D was higher than others. Besides, KRAS G12A mutated tumors had the likelihood of concurrent serine/threonine kinase 11 (STK11) and kelch-like ECH-associated protein 1 (KEAP1) mutations. KRAS G12C and tumor protein p53 (TP53) mutations could appear at the same time. In addition, KRAS G12D mutations and C-Ros oncogene 1 (ROS1) rearrangement were likely to be present in one tumor simultaneously. When the two drugs were combined, the respective IC50 values were lower than when used alone. In addition, there was a minimum number of clones among all wells in the drug combination. In in vivo experiments, the tumor size reduction in the drug combination group was more than twice that of the single drug group (*p* < 0.05). The differential expression genes were enriched in the pathways of phosphatidylinositol 3 kinase-protein kinase B (PI3K-Akt) signaling and extracellular matrix (ECM) proteoglycans compared the combination group to the control group.

**Conclusions:**

The anticancer effect of the drug combination was confirmed to be better than monotherapy in vitro and in vivo. The results of this study may provide some information for the plan of neoadjuvant therapy and the design of clinical trials for lung adenocarcinoma patients with KRAS G12C mutation.

**Supplementary Information:**

The online version contains supplementary material available at 10.1007/s12672-023-00698-z.

## Introduction

Lung cancer is still the leading cause of worldwide malignancies-related death, which mainly contains two histological types, small cell lung cancer and non-small cell lung cancer (NSCLC) [1, 2]. The proportion of NSCLC is over 80%, and the 5-year overall survival (OS) rate is less than 30% [3, 4]. Squamous cell carcinoma and lung adenocarcinoma account for most NSCLCs [5]. In addition, some somatic driver events were found to have the ability to provide treatment targets, such as epidermal growth factor receptor (EGFR) mutation, Kirsten rat sarcoma viral oncogene (KRAS) mutation, and CAP-Gly domain-containing linker protein 1- leukocyte tyrosine kinase (CLIP1-LTK) fusion [6–8]. Those findings improve the OS of NSCLC patients significantly [6, 7]. However, for patients of KRAS mutation, it is still a challenge to form the targeted drug because of the narrow hydrophobic pocket until some outstanding structural and mechanistic research constructed the theoretical basis for the clinical development of KRAS G12C inhibitors [9, 10].

Based on those findings, a phase 2 clinical trial (NCT03600883) of AMG-510 (Sotorasib) was performed in 126 enrolled patients. Moreover, the objective response rate reached 37.1% in this phase 2 trial [7]. According to the significant outcomes, the Food and Drug Administration approved the utilization of AMG-510 in NSCLC patients with KRAS G12C mutation as subsequent therapy [11]. Therefore, there is no doubt that the use of AMG-510 provides improved survival outcomes for NSCLC patients with KRAS G12C mutation. However, we found that some patients in this phase 2 trial did not have satisfactory outcomes—26 patients did not get disease control.

In addition, in a phase 2 trial of another targeted drug Erlotinib (EGFR inhibitor, NCT01407822), neoadjuvant targeted therapy did not further make tumor regression to elevate the complete resection rate and reduce resection extent [12]. Thus, the effect of tumor regression and the anticancer ability using monotherapy of targeted drugs are limited according to the results of the abovementioned studies [7, 12]. To consider those findings, some researchers investigated the antitumor effect of the drug combination in NSCLC patients with EGFR mutation and suggested that the EGFR inhibitor and chemotherapy combination further improved the objective response rate [13]. Therefore, this study aimed to explore whether the AMG-510 and cisplatin combination increases the antitumor effect in lung adenocarcinoma with the mutation of KRAS G12C by experiments in vitro and in vivo. In addition, we used bioinformatic analysis to uncover the potential mechanism of drug combination with improved anticancer effect. We anticipated that these results would provide some key information for designing clinical trials and using clinical drugs in the NSCLC population with KRAS G12C mutation.

## Methods

### Patients and samples

Patients with NSCLC after surgery were recruited from July 2018 to July 2019 in Sun Yat-sen University Cancer Center. Mian inclusion criteria for this study included: (1) histologically confirmed stage IA-IIIA NSCLC; (2) without distant metastasis; (3) without neoadjuvant therapy for NSCLC. Patients were excluded according to following standards: (1) age < 18 years old; (2) did not receive KRAS gene test. Tumor tissues which were collected during surgery were performed the next-generation sequencing (NGS). The pathological stage was based on the 8th staging system [14].

### Cell lines, cell culture, and drug treatment

Lung adenocarcinoma cell lines harbor KRAS G12C mutations were obtained from American Type Culture Collection. NCI-H23 (H23) and NCI-H358 (H358) cells were cultured in Roswell Park Memorial Institute 1640 medium (WISENT Inc., Nanjing, China) and supplemented with 10% fetal bovine serum (Excell Bio Inc., Taicang, China) and 50 µg/ml penicillin–streptomycin (Invitrogen). All cells used in this study were maintained in a 5% CO_2_ cell culture incubator at 37 °C. AMG-510 and Cisplatin (Targetmol Co. Ltd., Shanghai, China) were dissolved in dimethyl sulfoxide and H2O, respectively, then stored at -80 °C until use. Untreated cell lines were subdued until cell viability assays for determining the concentration inhibiting 50% of cell viability (IC50).

### Cell viability assay and IC50 determination

To determine the IC50 value of AMG-510 and Cisplatin in H23 and H358 cell lines, we used Cell Count Kit8 (CCK-8, Targetmol Co. Ltd., Shanghai, China) after 72 h of drug treatment. In this assay, 3000 cells were seeded on 96-well plates and incubated for cell apposition in their complete medium and subsequently for 72 h with scalar doses of AMG-510 (0–50 µM) or Cisplatin (0–5µM) as single agents or as a constant combination. After 72 h of drug treatment, we aspirated the old medium, added a new complete medium containing 10% CCK8 reagent, and placed it in a 37° thermostat for 2 h. It was worth noting that the process was kept dark throughout. Infinite M Plex (Tecan Trading Co., Ltd., Shanghai, China) and Tecan. At.XFluor software was used for signal acquisition with a detection wavelength of 450 nm. The dose-effect curves were drawn, and IC50 values were calculated by GraphPad Prism software version 9.0 (https://www.graphpad.com/) and analyzed by two-way ANOVA with Bonferroni’s correction.

### Colony formation

The cells were seeded into 6-well plates (2000 cells/well). Then medium changes were performed every other day, as well as medication changes. Each well was treated differently in the following four ways: control, 0.1µM AMG-510, 1µM Cisplatin, and 0.1µM AMG-510 plus 1µM Cisplatin. After 14–21 days of culture, the colonies were fixed with 4% paraformaldehyde and stained with 0.5% crystal violet (Yeasen Biotech Co., Ltd., Shanghai, China). The number of colonies was then counted.

### Mice experiments

**Fig. 1:**
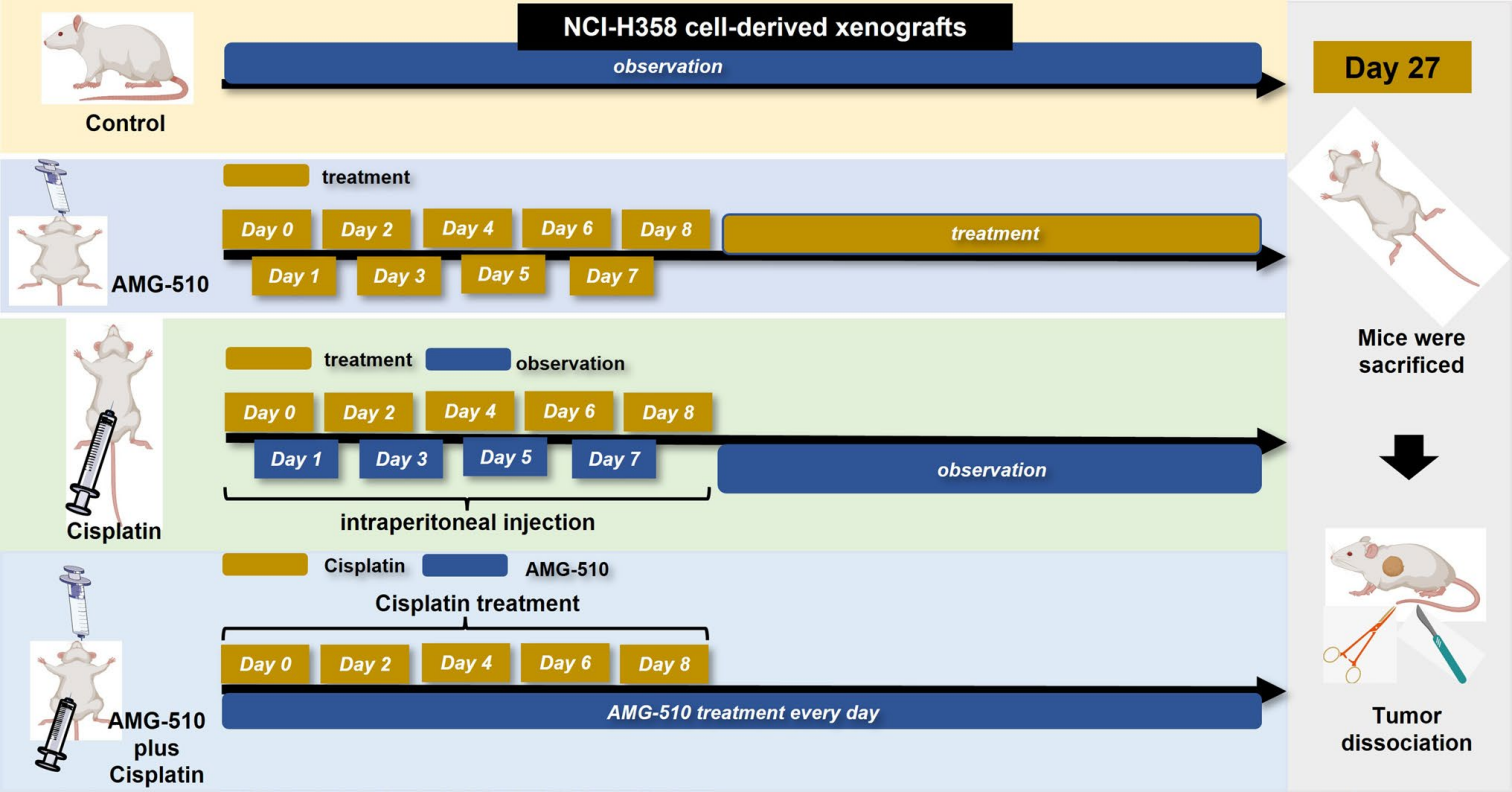
Flow chart of animal experiments

We constructed cell-derived xenografts (CDXs) to explore the anti-tumor effect of AMG-510, Cisplatin, and their combination in Balb/C nude mice (Yaokang Biotechnology Co., Ltd, Guangzhou, China, License No. SCXX2020-0054). The H358 cells (2*10^6^/150µL) were injected under the skin after one-week feeding. The mice were classified into four groups randomly (control, AMG-510, Cisplatin, and combination) when the tumor size was about 53-111mm^3^. Mice in the control group did not receive any drug; mice in the AMG-510 group were administered by gavage at a dose of 30 mg/kg per day (100 µl suspension); mice in the cisplatin group were administered by intraperitoneal injection at 4 mg/kg per mouse on alternate days, with follow-up observation after five injections; the administration of the combination drug group combined the AMG-510 and cisplatin groups. The specific dosing flow chart is shown in Fig. 1. The 0.5% sodium carboxymethylcellulose (Targetmol Co. Ltd., Shanghai, China) was used as the resuspension medium for AMG-510, forming a stable homogeneous mixture. Cisplatin was dissolved in 0.9% saline into a working solution of 4 mg/kg. Mice were weighed regularly. After 28 days from the first drug injection observation, the difference in tumor size between the four groups was evident, and the animal experiment was ended. After anesthetizing the mice, the mice were executed using the cervical dislocation method, then the tumors were dissected, the tumors were weighed, and the tumor tissue was preserved.

### mRNA sequence

Total RNA was extracted using the TRIzol reagent (Invitrogen, CA, USA) according to the manufacturer’s protocol. In this study, we used resected tumor tissue to perform this experiment. RNA purity and quantification were evaluated using the NanoDrop 2000 spectrophotometer (Thermo Scientific, USA). RNA integrity was assessed using the Agilent 2100 Bioanalyzer (Agilent Technologies, Santa Clara, CA, USA). Then the libraries were constructed using VAHTS Universal V6 RNA-seq Library Prep Kit according to the manufacturer’s instructions.

The libraries were sequenced on a llumina Novaseq 6000 platform and 150 bp paired-end reads were generated. About 50 M raw reads for each sample were generated. Raw reads of fastq format were firstly processed using fastp and the low-quality reads were removed to obtain the clean reads [15]. Then about 40 M clean reads for each sample were retained for subsequent analyses. Finally, the clean reads were mapped to the reference genome using HISAT2 [16].

### Statistical analysis

Fragments Per Kilobase of exon model per Million mapped fragments (FPKM) of each gene were calculated, and the read counts of each gene were obtained by HTSeq-count [17, 18]. Principal component analysis (PCA) analysis was performed using R 4.2.2 software (https://cloud.r-project.org/) to evaluate the biological duplication of samples. Differential expression analysis was performed using the DESeq2 [19]. Q value < 0.05 and foldchange > 2 or foldchange < 0.5 was set as the threshold for significantly differential expression genes (DEGs). Hierarchical cluster analysis of DEGs was performed using R 4.2.2 software to demonstrate the expression pattern of genes in different groups and samples. The radar map of the top 30 genes was drawn to show the expression of up-regulated or down-regulated DEGs using the R package “ggradar.” Based on the hypergeometric distribution, Gene Ontology (GO), Kyoto Encyclopedia of Genes and Genomes (KEGG), Wikipathway, and Reactome pathway enrichment analysis of DEGs were performed to screen the significantly enriched term using R 4.2.2 software, respectively [20–22]. The GO entries corresponding to the number of differential genes greater than 2 in the three classifications (biological process [BP], cellular component [CC], and molecular function [MF]) were screened, and ten entries were sorted by the -log10p-value of each entry in descending order. R 4.2.2 software was used to draw the column, chord, and bubble diagram of the significant enrichment term. Gene Set Enrichment Analysis (GSEA) was performed using GSEA software. Besides, we used the STRING database to conduct Protein-Protein Interaction Networks (PPI) analysis and screened the top 30 DEGs with a high combined score [23]. The analysis used a predefined gene set, and the genes were ranked according to the degree of differential expression in the two samples. Then, whether the predefined gene set was enriched at the top or bottom of the ranking list is tested. The Mann-Whitney U test was used to compare the difference of tumor weight between two groups. The standard deviation (SD) was conducted to evaluate the stability of data. A two-sided p-value < 0.05 was considered statistically significant.

## Results

### Patient characteristics

We reviewed the medical records of 495 NSCLC patients. The proportion of KRAS mutation was 2.2% (11/495). Those patients underwent surgical resection, and the majority of them belonged to stage IA disease (63.6%, 7/11). Two patients developed tumor recurrence at postoperative month three and month 12. In this cohort with KRAS mutation, the proportion of G12D was higher than others. Besides, KRAS G12A mutated tumors had the likelihood of concurrent serine/threonine kinase 11 (STK11) and kelch-like ECH-associated protein 1 (KEAP1) mutations. KRAS G12C and tumor protein p53 (TP53) mutations could appear at the same time. In addition, KRAS G12D mutations and C-Ros oncogene 1 (ROS1) rearrangement were likely to be present in one tumor at the same time. The detailed clinical characteristics are shown in Table 1.

**Table 1:**
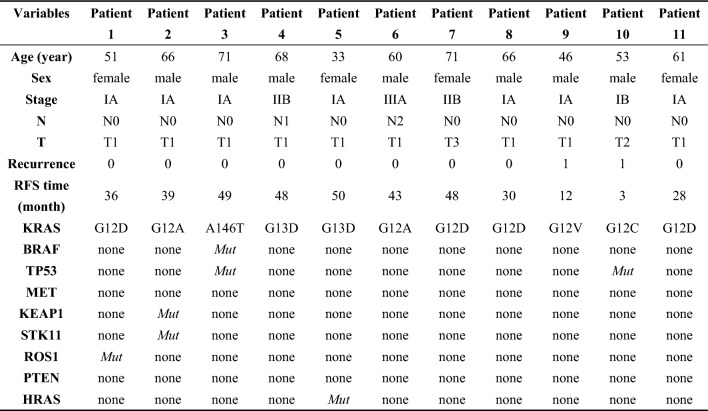
Clinical characteristics of non-small-cell lung cancer patients with KRAS mutation

*RFS* recurrence-free survival; *BRAF* vrafmurine sarcoma viral oncegene homolog B; *TP53* tumor protein p53; *MET*: mesenchymal epithelial transition factor; *KEAP1*: Kelch-1ike ECH- associated protein l; *STK11*: serine/threonine Kinase 11; *ROS1*: ROS proto-oncogene 1; *PTEN* phosphatase and tensin homolog; *HRAS* v-Ha-ras Harvey rat sarcoma viral oncogene homolog; *Mut*: mutation

### Cell viability and colony formation

**Fig. 2:**
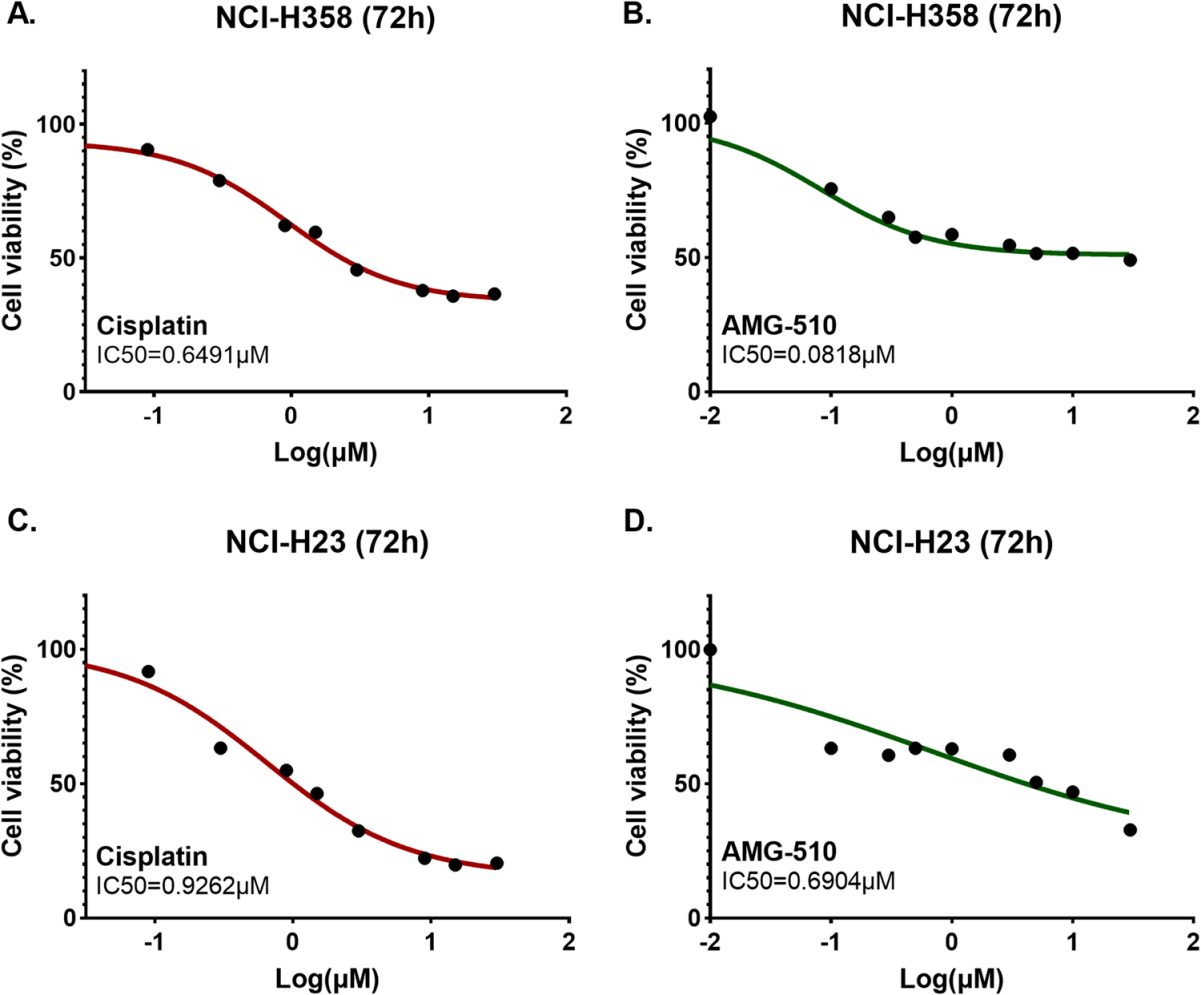
Cell viability experiment for monotherapy in different cell lines

**Fig. 3:**
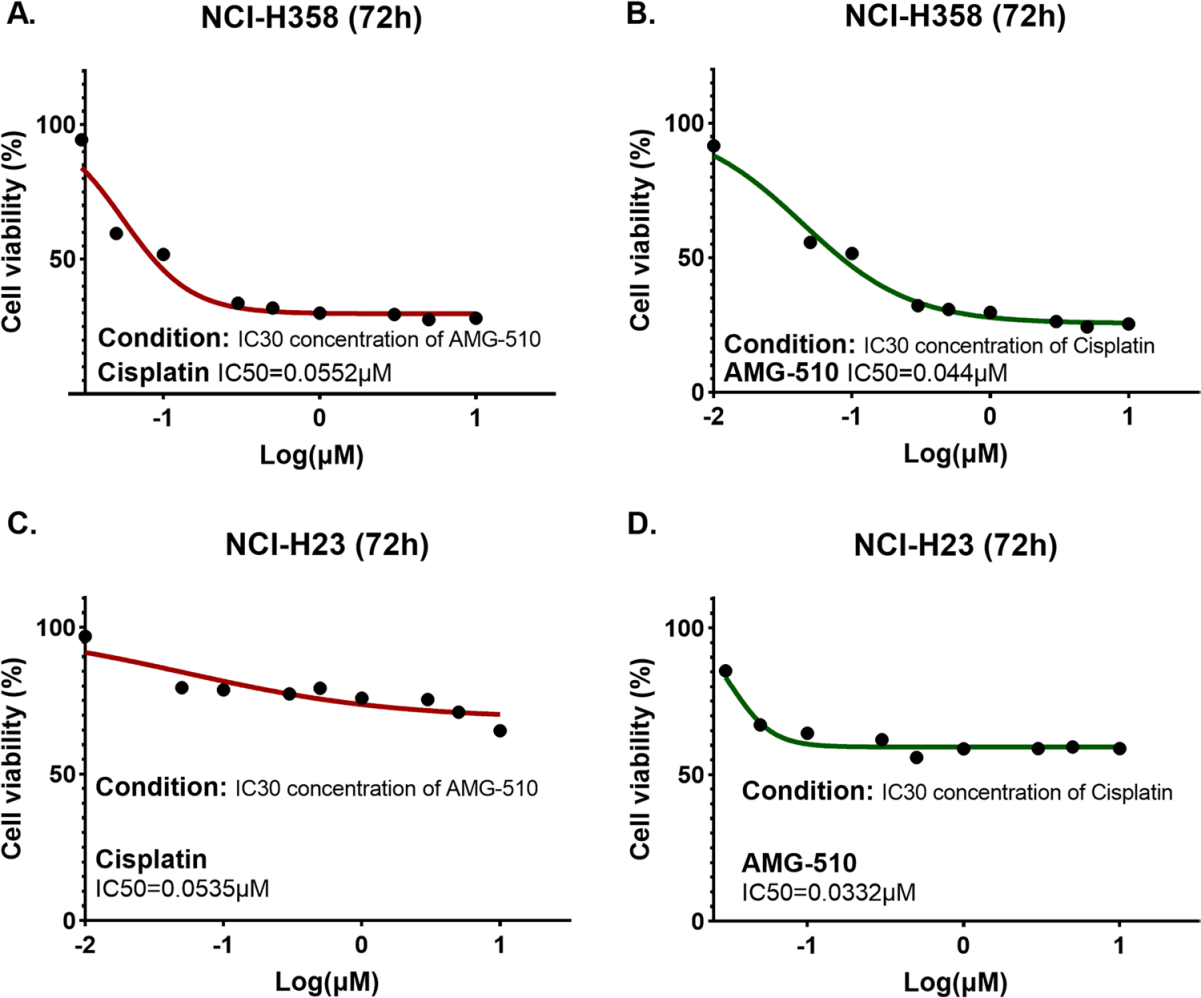
Cell viability experiment for drug combination in different cell lines

**Fig. 4:**
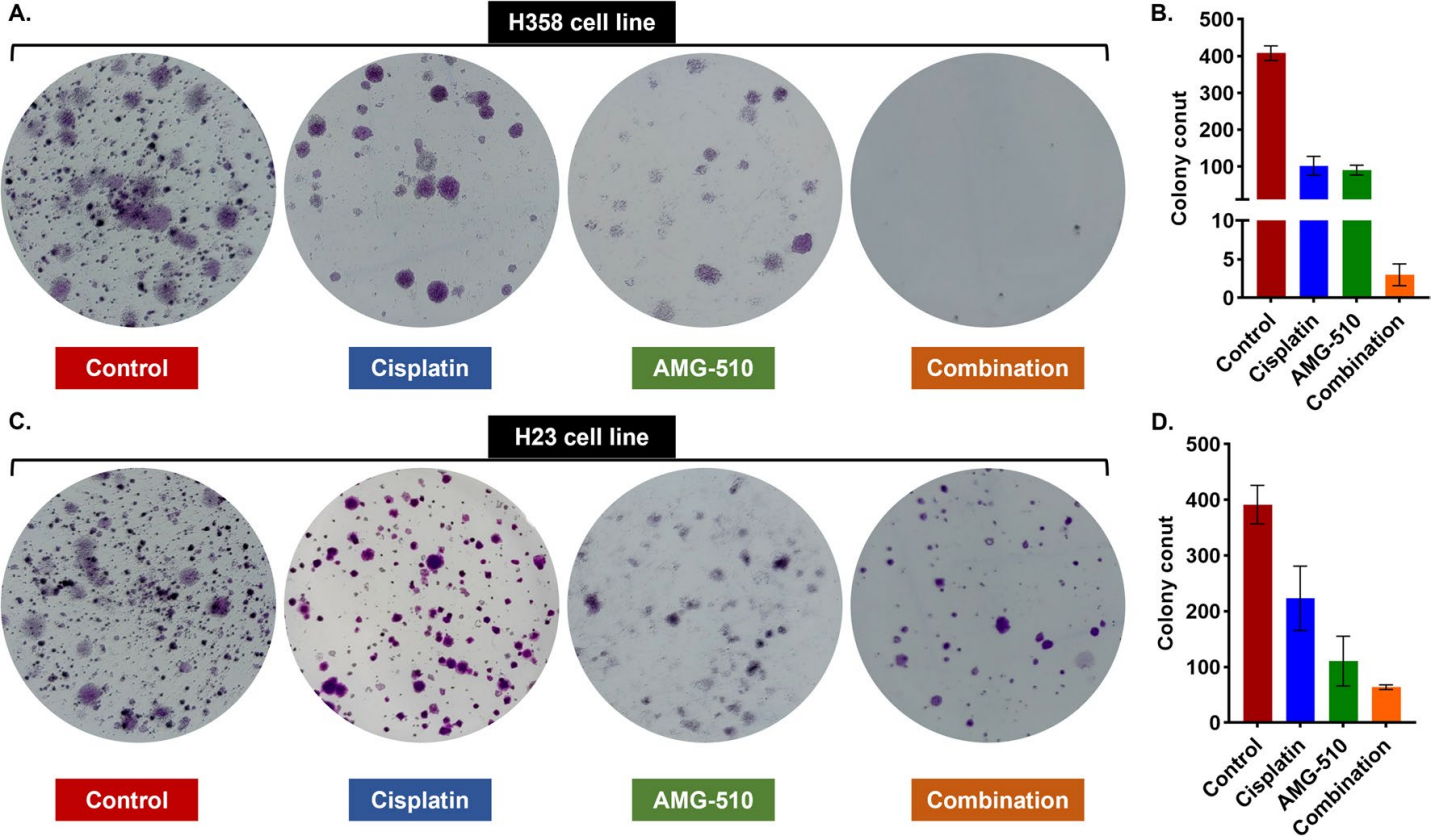
Colony formation experiment and colony count for H358 (**A** and **B**) and H23 (**C** and **D**) cell lines

In the cell viability experiments, H358 and H23 cell lines were sensitive to drug treatment. The IC50 values of Cisplatin and AMG-510 in H358 and H23 cell lines were 0.6491µM vs. 0.0818µM and 0.9262µM vs. 0.6904µM, respectively (Fig. 2). To explore the anticancer effect of combination with two drugs in both cell lines, we fixed the concentration of one drug (setting the concentration of IC30) and increased the concentration of the other drug in a gradient. The IC30 concentration of Cisplatin in H358 and H23 cell lines were 0.27µM and 0.216µM, respectively. Besides, the IC30 concentration of AMG-510 in H358 and H23 cell lines were 0.035µM and 0.044µM, respectively. Under the condition with the IC30 concentration of AMG-510 pre-treatment, the IC50 values of Cisplatin were 0.0552µM and 0.0535µM in the H358 and H23 cell lines (Fig. 3). Similarly, with the combination of IC30-concentration Cisplatin, the IC50 values of AMG-510 were 0.044µM and 0.0332µM in the H358 and H23 cell lines (Fig. 3). Overall, when the two drugs were combined, the respective IC50 values were lower than when the drugs were used alone. As showed results in Fig. 4, the inhibition effect of drugs presents an increasing trend. The combination of AMG-510 and Cisplatin had the best anticancer ability among different use of drugs. In addition, in wells of the drug combination, there was the minimum number of clones among all wells (Fig. 4B and D).

### Confirmation of anticancer effect in vivo

**Fig. 5:**
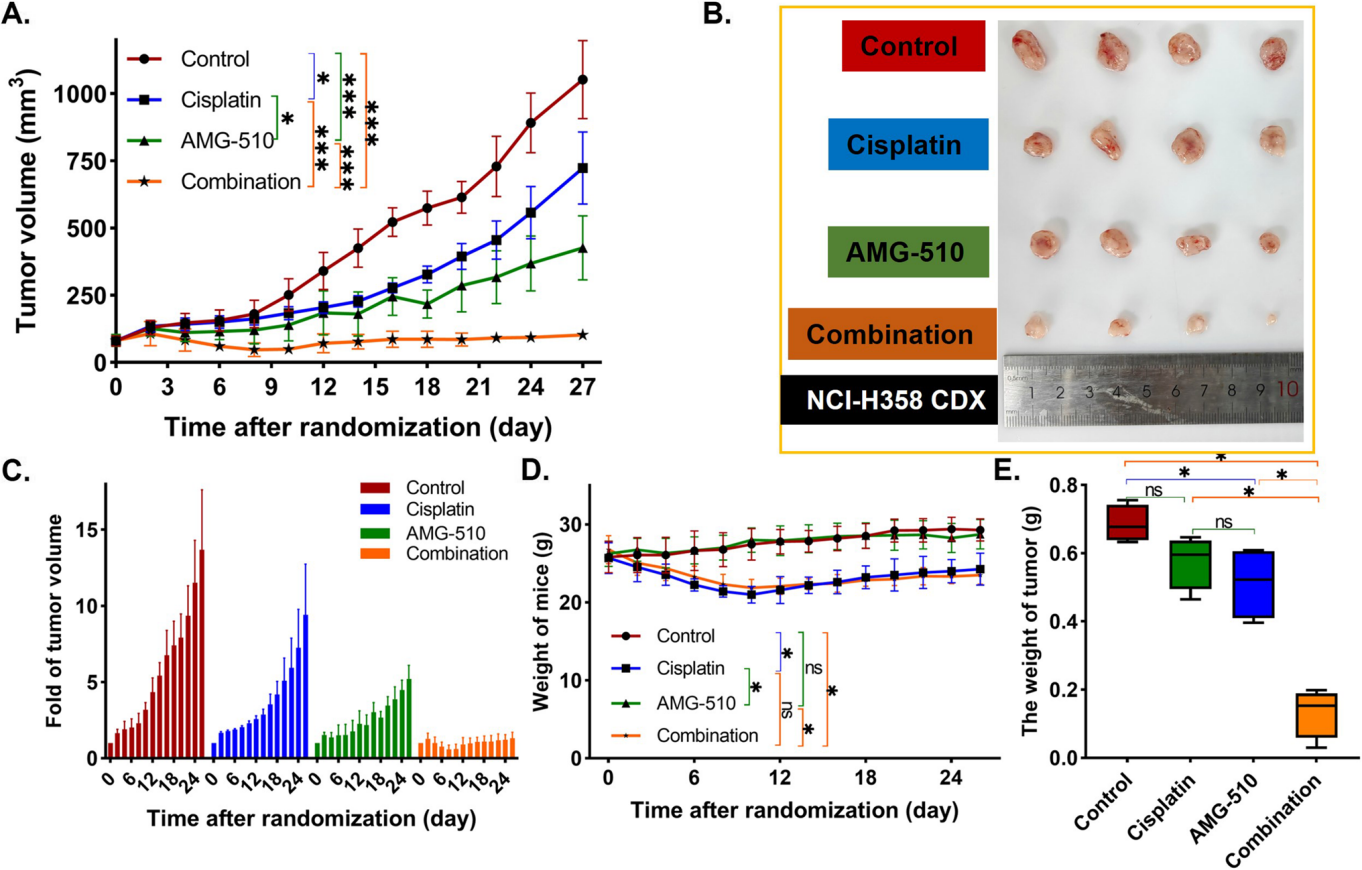
The growth of the tumor volume over time (**A**). The photo of tumor removed from mice (**B**). The fold change of tumor volume over time (**C**). The weight of mice over time (**D**). The wight of tumor in the end (**E**)

Animal experiments were performed to validate the anticancer effect of the drug combination in vivo. The flow chart is presented in Fig. 1. After the tumors were measurable, we randomized the mice to groups. Animal experiments were performed for 28 days, and the changes in tumor volume in each group can be seen in Fig. 5A, B. The mean tumor volume were 1052.56mm^3^ (SD = 142.54mm^3^), 723.39mm^3^ (SD = 133.88mm^3^), 426.66mm^3^ (SD = 118.72mm^3^), and 102.51mm^3^ (SD = 20.14mm^3^) in control, Cisplatin, AMG-510, and combination groups on Day 27, respectively. The size reduction of tumor in the drug combination group was more than twice that of the single drug group. At the end of the animal experiments, the tumor volume in each group was the smallest in the combined drug group (AMG-510 group vs. combination group, *p* < 0.001; Cisplatin group vs. combination group, *p* < 0.001). The picture to fold of tumor volume showed that the tumor growth in the combination group was evidently inhibited (Fig. 5C). Besides, the mean weight of mice was 29.28 g (SD = 1.42 g), 24.25 g (SD = 2.05 g), 28.73 g (SD = 1.87 g), and 23.50 g (SD = 1.17 g) in control, Cisplatin, AMG-510, and combination groups on Day 27, respectively. Compared to the control and AMG-510 groups, Cisplatin and combination groups had more severe weight loss (all *p* < 0.05, Fig. 5D). Overall, however, the weight of the combination group remained comparable to that of the Cisplatin group (*p* = 0.549). Among control (0.69 g, SD = 0.05 g), Cisplatin (0.58 g, SD = 0.08 g), AMG-510 (0.51 g, SD = 0.11 g), and combination groups (0.13 g, SD = 0.07 g), the tumor weight was the smallest in the combination groups (Fig. 5E).

### The landscape of DEGs

**Fig. 6:**
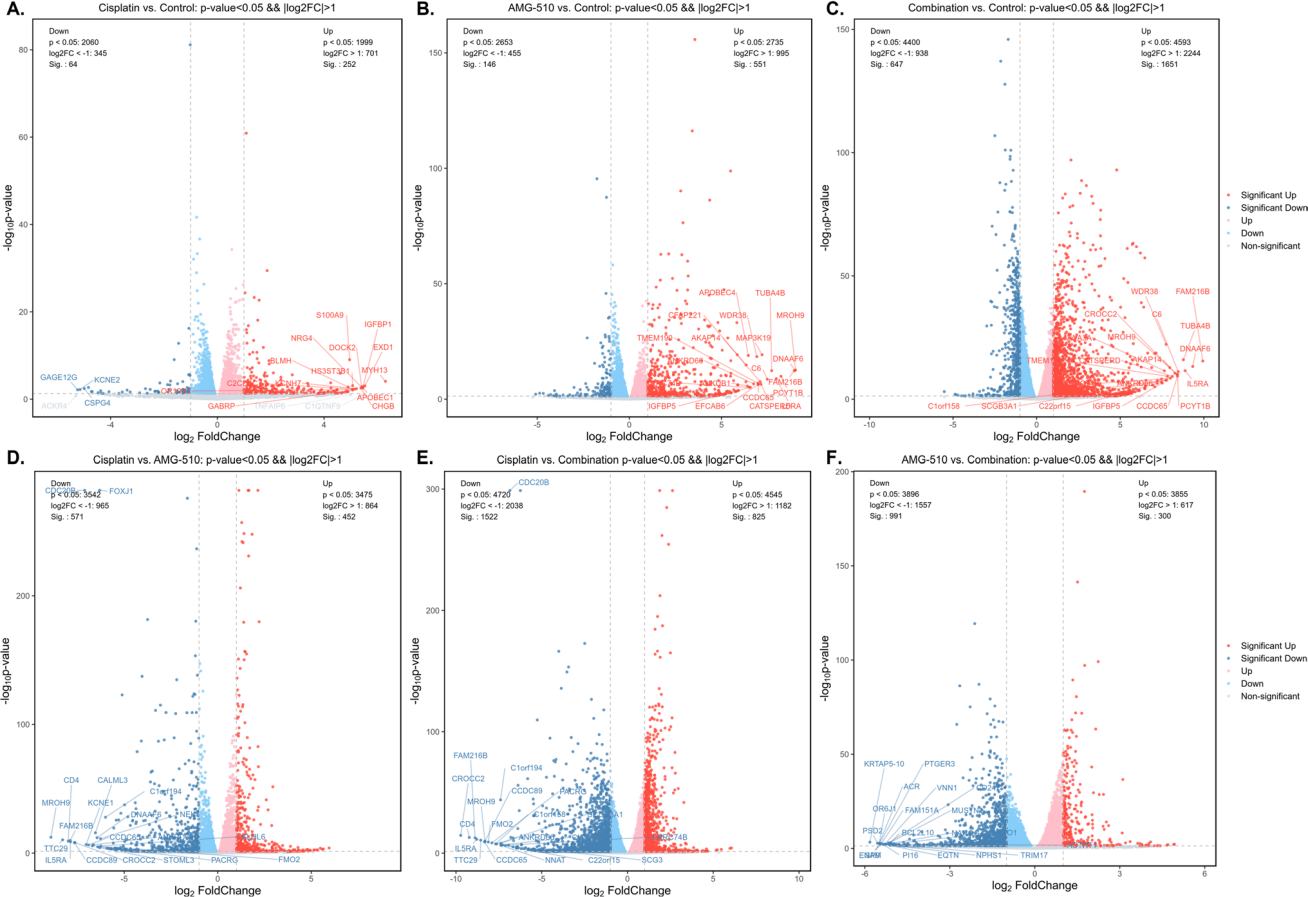
The differential expressed genes (DEGs) in different comparisons

**Fig. 7:**
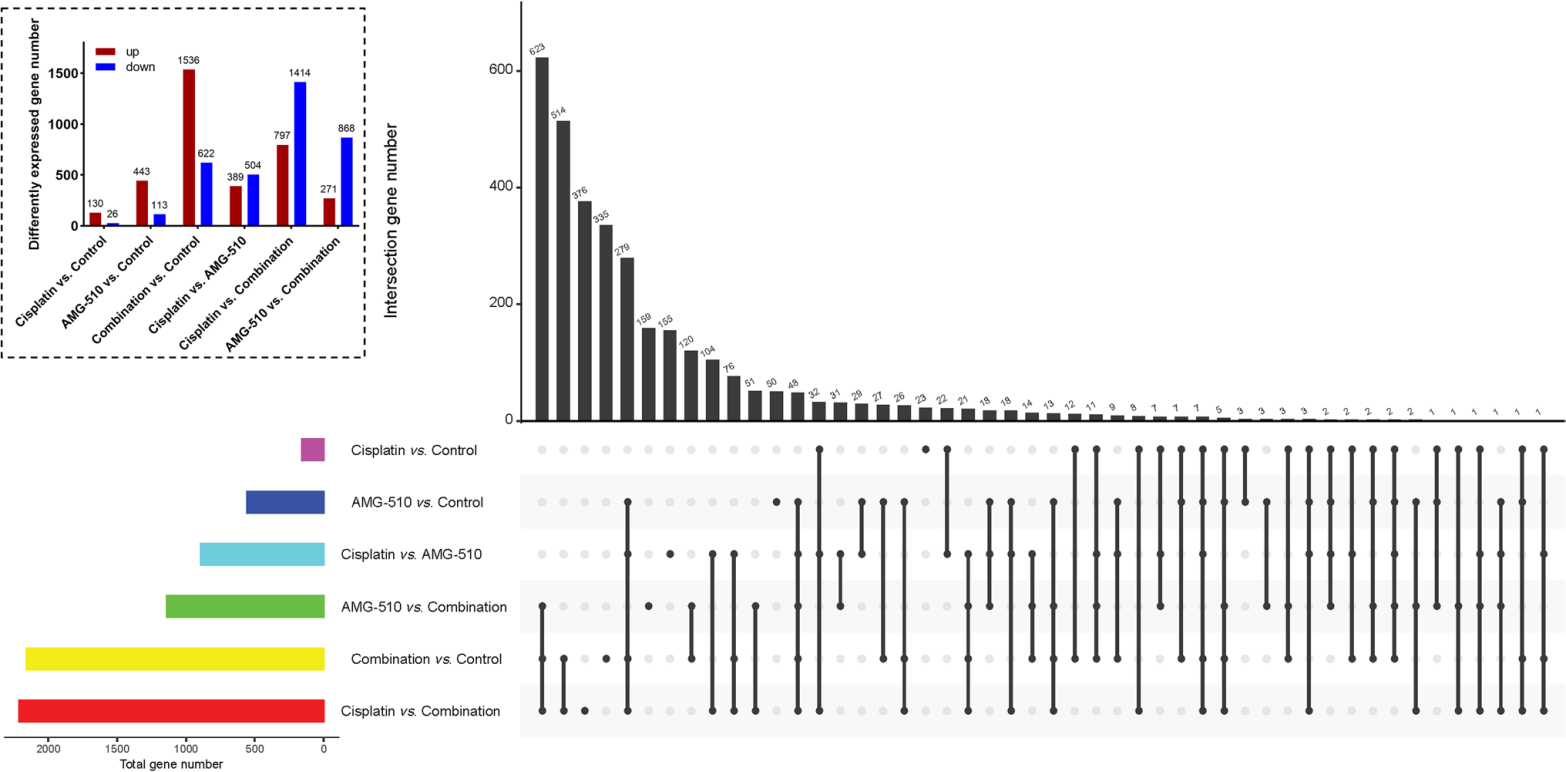
The number of the differential expressed genes (DEGs) in different comparisons

After differential expression analysis in the data of RNA seq among resected tumor tissues from mice of control, Cisplatin, AMG-510, and combination groups, up-regulated and downregulated DEGs were obtained. In the different comparison groups, the DEGs were different. Detailed information about DEGs could see in Figs. 6 and 7. There were 504 down-regulated and 389 up-regulated DEGs in the comparison between Cisplatin and AMG-510 (Figs. 6D and 7). Besides, in comparing the Cisplatin and Combination, 797 up-regulated genes and 1414 down-regulated genes were demonstrated (Fig. 6E). As for AMG-510 vs. Combination, 271 up-regulated and 868 down-regulated were included in this analysis (Fig. 6F).

### Functional enrichment and PPI analyses of DEGs

**Fig. 8:**
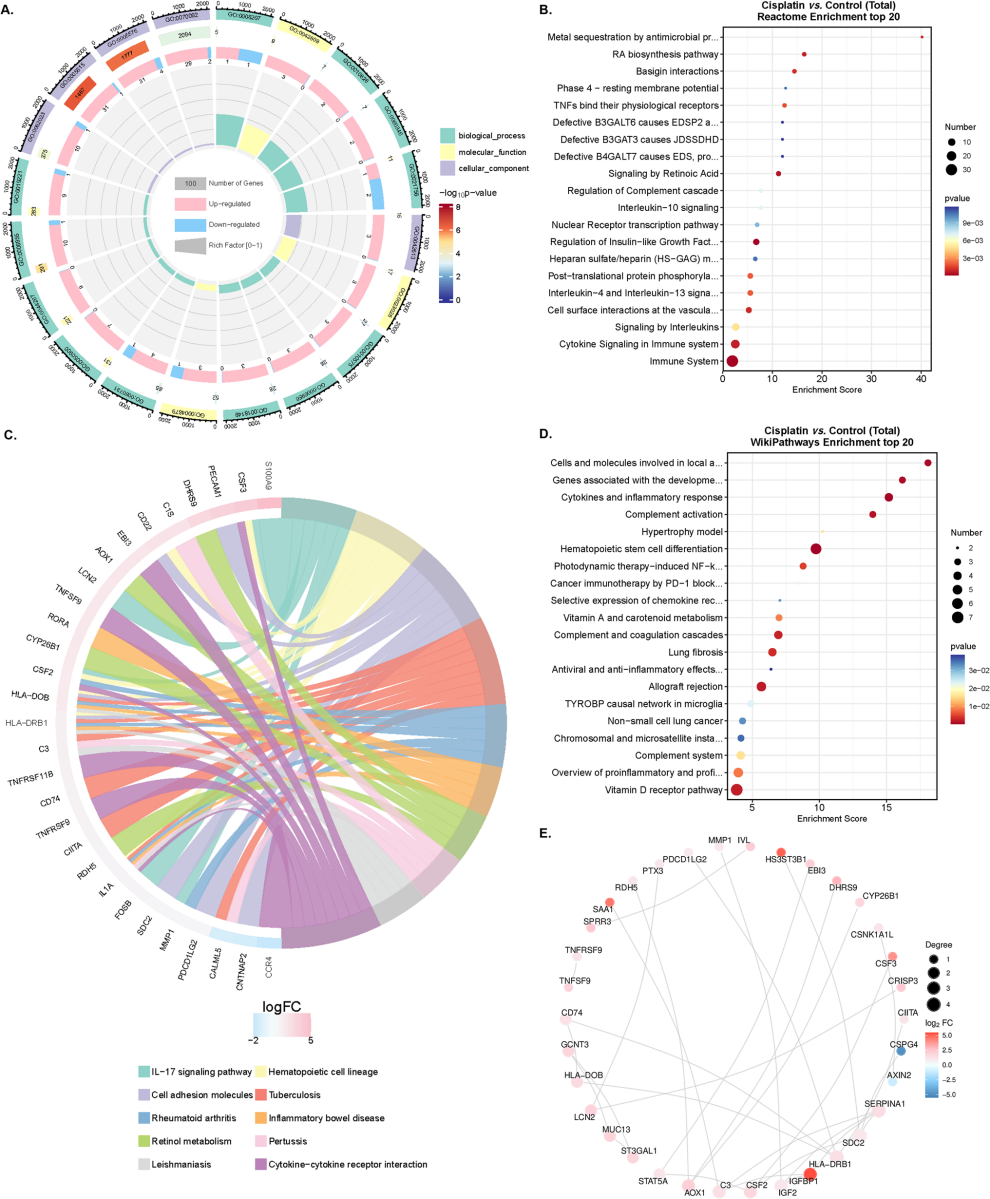
The GO enrichment (**A**), Reactome enrichment (**B**), KEGG pathway (**C**), WikiPathway (**D**), and Protein-Protein Interaction Networks (**E**) analyses in the comparison between Cisplatin and Control groups

To study the relationship among DEGs and BPs, MFs, CCs, biological pathways, and diseases in comparing the Cisplatin and Control group, we first performed a functional enrichment analysis of DEGs (Fig. 8A). DEGs were the most abundant in BPs, such as biological adhesion, cell killing, and immune system processing (Supplementary Figure 1A). Further, the DEGs obtained in the comparison of Cisplatin vs. control was enriched in the pathway of the immune system after KEGG and Reactome analyses (Supplementary Figure 1B and Fig. 8B–C). Besides, the pathways of hematopoietic stem cells, cytokines, and inflammatory response were founded in the DEGs enrichment according to the results of Wikipathway analysis (Fig. 8D). According to the analysis of PPI, serum amyloid A1 (SAA1), heparan sulfate 3-O-sulfotransferase 3B1 (HS3ST3B1), and insulin-like growth factor binding protein 1 (IFGBP1), as up-regulated DEGs, had more related genes than other genes (Fig. 8E).

**Fig. 9:**
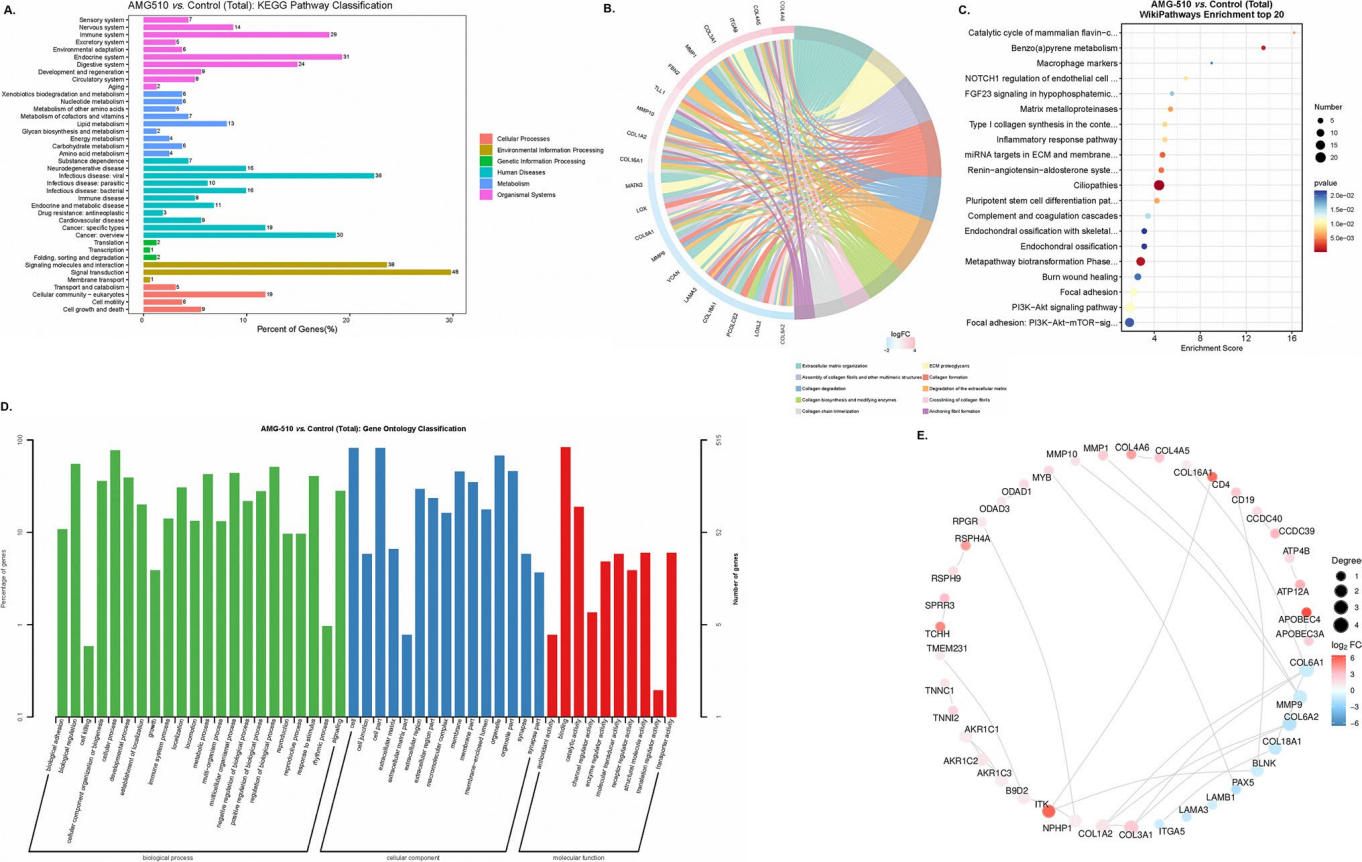
The KEGG pathway (**A**), Reactome enrichment (**B**), WikiPathway (**C**), GO enrichment (**D**), and Protein-Protein Interaction Networks (**E**) analyses in the comparison between AMG-510 and Control groups

For the comparison between AMG-510 vs. control, the DEGs were enriched in the pathways of signal transduction and signaling molecules and interaction in the KEGG analysis (Fig. 9A). Besides, Reactome analysis showed there were many DEGs enriched in the pathway of extracellular matrix organization (Fig. 9B). After WikiPathway analysis, the results presented that the ciliopathies gathered many DEGs (Fig. 9C). GO enrichment analysis suggested that the DEGs were rich in the BP function (Fig. 9D). In addition, the PPI analysis suggested that four down-regulated DEGs (collagen, type VI, alpha 1 [COL6A1], matrix metallopeptidase 9 [MMP9], collagen, type VI, alpha 2 [COL6A2], and B-cell linker protein [BLNK]) had the largest number of related genes among all DEGs (Fig. 9E).

**Fig. 10:**
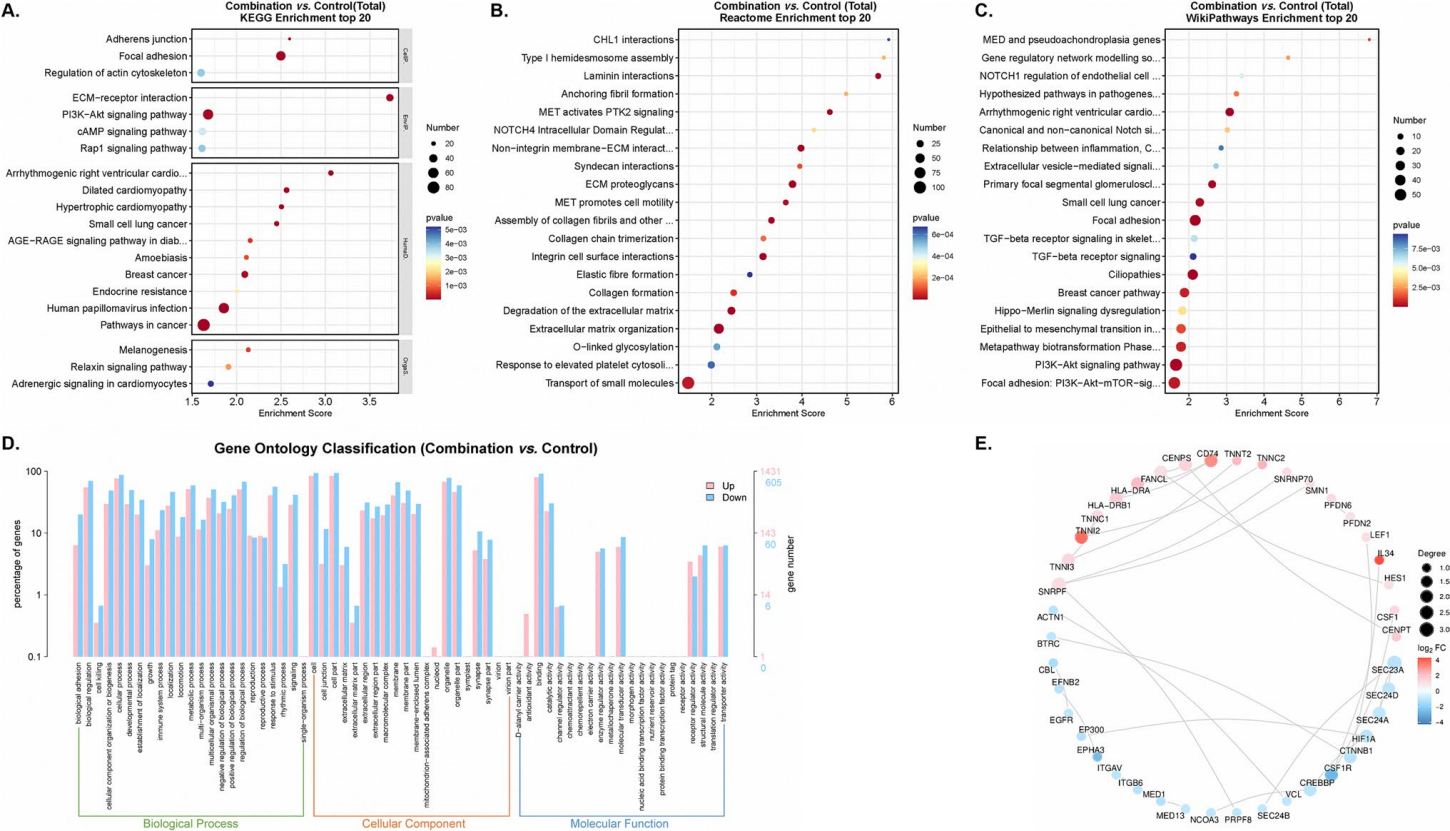
The KEGG pathway (**A**), Reactome enrichment (**B**), WikiPathway (**C**), GO enrichment (**D**), and Protein-Protein Interaction Networks (**E**) analyses in the comparison between combination and Control groups

We further focused on the DEGs obtained in the comparison between the combination and control groups. We found that the DEGs were enriched in the pathways of phosphatidylinositol 3 kinase-protein kinase B (PI3K-Akt) signaling and extracellular matrix (ECM) proteoglycans after analyses of KEGG enrichment, Reactome enrichment, and WikiPathway (Fig. 10A–C). GO classification uncovered that the DEGs were mainly located in the BP function, and were enriched in the cilium movement, cell adhesion, and calcium ion binding function pathways (Fig. 10D). Of note, the up-regulated DEGs were different from the abovementioned two comparisons, of which three DEGs (CD74 molecule [CD74], troponin I2, fast skeletal type [TNNI2], and interleukin 34 [IL34]) had the most number of related genes among genes (Fig. 10E).

## Discussion

In this study, we used the data from our hospital to explore the proportion of KRAS in all patients and found that KRAS G12C and TP53 mutations could appear at the same time, and KRAS G12D mutations and ROS1 rearrangement were likely to be present in one tumor at the same time. Besides, we explored the anticancer effect of the combination of Cisplatin and AMG-510 and suggested that the combination treatment had a better ability to regress the tumor than the use of the single drug in vitro and in vivo (Figs. 3, 4 and 5). To further explore the possible mechanism of the good effect of the dual drug combination, we used some methods of bioinformatics for the analysis of the RNA-seq data. In addition, we found that the DEGs obtained in the comparison between the combination and control groups were enriched in the pathways of PI3K-Akt signaling, cilium movement, cell adhesion, and calcium ion binding function pathways and ECM proteoglycans after analyses of KEGG enrichment, Reactome enrichment, GO classification, and WikiPathway (Fig. 10). Interestingly, CD74, TNNI2, and IL34, as the up-regulated DEGs, had the largest number of related genes in all DEGs (Fig. 10E).

Neoadjuvant therapy is essential for local advanced NSCLC patients. The mood of preoperative drug treatment plus surgical resection provides more survival benefits than surgery or drug treatment alone in those patients [24]. Accordingly, a number of clinical trials focused on NSCLC patients with locally advanced staging disease [25, 26]. In a previous study, however, the use of targeted therapy alone did not increase the rate of R0 (complete resection) compared with chemotherapy. Even more, the data showed that patients treated with targeted agents had (8.1%, n = 3) a higher rate of R2 resection (visible tumor under the naked eye) than those treated with chemotherapy (2.9%, n = 1) [12]. R2 resection means that patients have higher recurrence rates and shorter disease-free survival. Thus, it is important to increase the rate of tumor regression for patients with locally advanced staging disease. As for translational and preclinical research, the results of this study may provide some information for the plan of neoadjuvant therapy and the design of clinical trials for NSCLC patients with KRAS G12C mutation. First, the anticancer effect of the use of drug combination was confirmed to be better than monotherapy in vitro and in vivo. Interestingly, the reduction of tumor size in the drug combination group was more than twice that of the single drug group. Besides, as seen in Fig. 5 of the fold of tumor volume, the tumors in the control group showed a growing trend over time, and the monotherapy group only inhibited the growing trend, while the combination group interrupted the tumor growth trend. Therefore, the combination of AMG-510 and Cisplatin may be a good trial for KRAS G12C mutation patients with locally advanced staging disease in phase I/II clinical trials. Second, although the weights of the mice did not differ between the cisplatin and combination groups, one of the mice in the combination group died during the first cycle of Cisplatin. Accordingly, it is still important to pay attention to the side effects during the drug combination, especially the tolerance of patients during the first cycle of chemotherapy and targeted drugs.

The proportion of KRAS G12C mutation is varied in NSCLC patients among different cohorts. A previous report suggested that among NSCLC patients, White (13.0%) and Black patients (10.9%) had more proportion of KRAS G12C mutations than Asians (3.6%) [27]. Other studies also confirmed this distribution phenomenon of KRAS G12C mutation [28–30]. The proportion of KRAS G12C mutation was low in the present study, like the abovementioned research. Thus, the low mutation rate of KRAS G12C in Asian populations reminds to researchers to consider the proper use of resources and fit when conducting research. Besides, as reported in previous studies, our study also found that STK11 can be co-mutated with KRAS G12A [31]. Interestingly, we found that ROS1 rearrangements could coexist with KRAS G12D, which was not common in other studies. Although in our study, two patients with KRAS G12C and KRAS G12V recurred, because of the small sample size, it cannot be said that patients with these two mutations have a poor prognosis. It’s essential for NSCLC patients to perform gene tests because exact gene status means personalized medicine and survival benefit [32]. Recently, a phase III clinical trial (NCT04303780) uncovered that AMG-510 significantly increased progression-free survival compared with docetaxel in patients with advanced NSCLC with the KRAS G12C mutation [33]. Patients need to be confirmed for KRAS G12C mutation prior to AMG-510 treatment. Therefore, molecular testing is necessary for the design of clinical trials.

The potential mechanism of the anticancer effect from a combination of AMG-510 and Cisplatin is complex, and it is not a simple superposition of the cancer-inhibiting mechanisms of chemotherapeutic agents or AMG-510. The DEGs differed among three comparisons (Fig. 6, Cisplatin vs. Control, AMG-510 vs. Control, and Combination vs. Control). Accordingly, the DEGs enrichment’s function pathway was inconsistent (Figs. 8, 9 and 10). After Cisplatin treatment, the DEGs were mainly located in the pathway of immune system. Besides, the use of AMG-510 inhibited the expression level of MMP9, which could promote tumor metastasis through the PI3K/Akt pathway [34]. The DEGs obtained from the comparison between AMG-510 and control groups were enriched in the function pathway of signal transduction. The extracellular matrix (ECM) pathway-related genes, like COL16A1 and COL4A6, were up-regulated after AMG-510 treatment. Notably, the ECM pathway may be related to tumor progression and drug resistance [35]. In addition, the phenomenon that the DEGs were enriched in the pathway of PI3K-Akt after treatment combination of AMG-510 and Cisplatin may indicate one of the drug resistance mechanisms.

This study still has some shortcomings. First, we only performed simple, functional experiments and did not further investigate the mechanism. However, the functional validation experiments have provided useful information for conducting subsequent clinical trials of neoadjuvant therapy. Second, the clinical data in this study were partially from a single hospital with a small sample size, and further expansion of the sample size is still needed to confirm our findings. Third, because the drug AMG-510 is not yet available in China, we could not obtain samples from patients after dosing them for genetic testing. We hope that AMG-510 will be available in China soon to benefit more lung cancer patients.

## Conclusions

The anticancer effect of the use of drug combination was confirmed to be better than monotherapy in vitro and in vivo. The results of this study may provide some information for the plan of neoadjuvant therapy and the design of clinical trials for lung adenocarcinoma patients with KRAS G12C mutation.

## Supplementary Information

Supplementary material 1: The GO enrichmentand KEGG pathwayclassifications.

## Data Availability

Any researchers interested in this study could contact corresponding author for requiring data.
