# Supplementary figures and images for: AMG-510 and cisplatin combination increases antitumor effect in lung adenocarcinoma with mutation of KRAS G12C: a preclinical and translational research
Source: Discov Oncol. 2023 Jun 7;14:91. doi: 10.1007/s12672-023-00698-z (PMC10247598; doi:10.1007/s12672-023-00698-z)

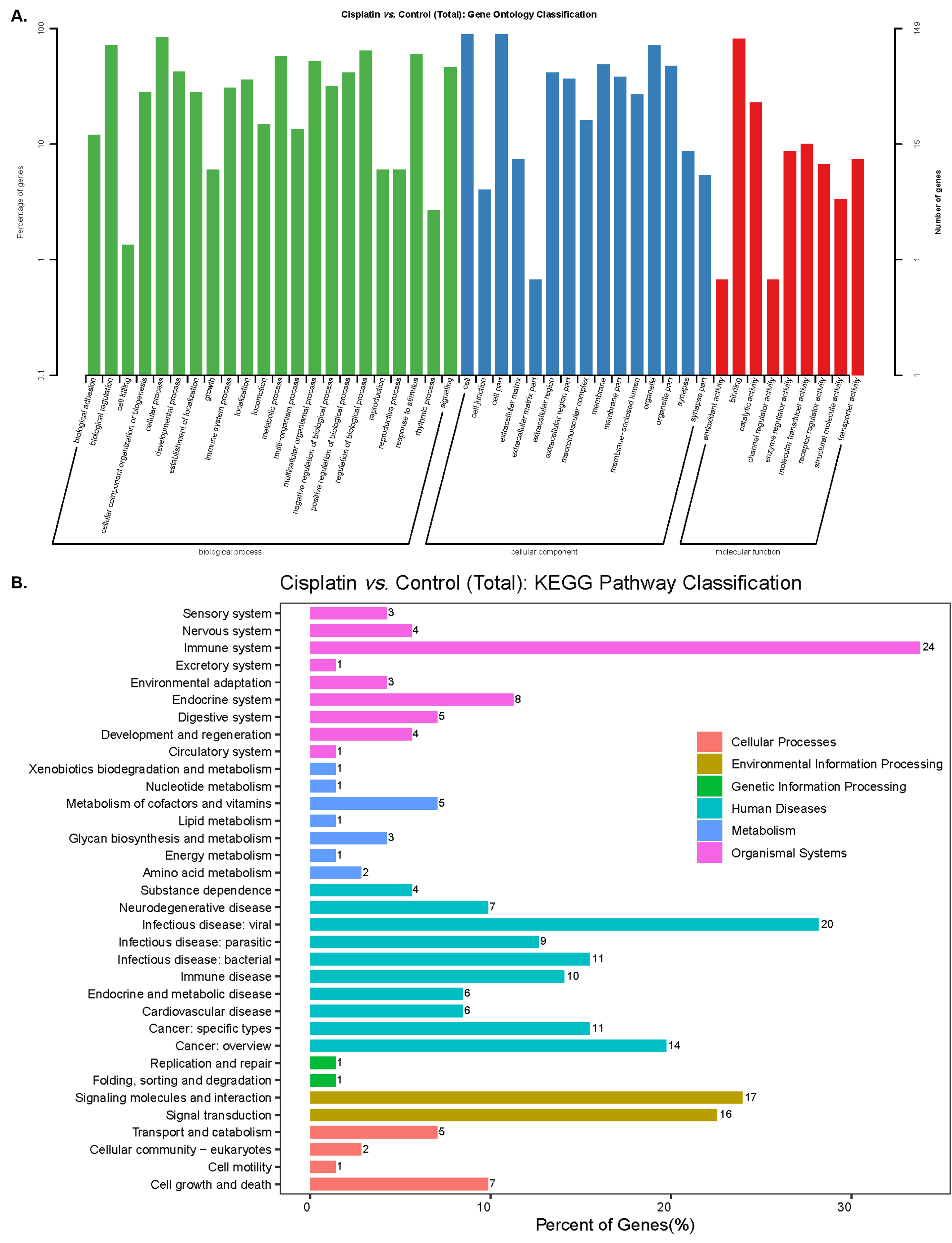

Supplement: Supplementary file 1 — Supplementary material 1: The GO enrichmentand KEGG pathwayclassifications. [file 12672_2023_698_MOESM1_ESM.png]
